# Genetics of frontotemporal lobar degeneration

**DOI:** 10.4103/0972-2327.74246

**Published:** 2010-12

**Authors:** P. M. Aswathy, P. S. Jairani, P. S. Mathuranath

**Affiliations:** Cognition & Behavioural Neurology Section (CBNS), Department of Neurology, Sree Chitra Tirunal Institute for Medical Sciences & Technology (SCTIMST), Thiruvananthapuram, Kerala, India

**Keywords:** Frontotemporal lobar degeneration, genetic risk factors, microtubule-associated protein tau, mutations, progranulin, TDP-43

## Abstract

Frontotemporal lobar degeneration (FTLD) is a highly heterogenous group of progressive neurodegenerative disorders characterized by atrophy of prefrontal and anterior temporal cortices. Recently, the research in the field of FTLD has gained increased attention due to the clinical, neuropathological, and genetic heterogeneity and has increased our understanding of the disease pathogenesis. FTLD is a genetically complex disorder. It has a strong genetic basis and 50% of patients show a positive family history for FTLD. Linkage studies have revealed seven chromosomal loci and a number of genes including *MAPT, PGRN, VCP*, and *CHMB-2B* are associated with the disease. Neuropathologically, FTLD is classified into tauopathies and ubiquitinopathies. The vast majority of FTLD cases are characterized by pathological accumulation of tau or TDP-43 positive inclusions, each as an outcome of mutations in *MAPT* or *PGRN*, respectively. Identification of novel proteins involved in the pathophysiology of the disease, such as progranulin and TDP-43, may prove to be excellent biomarkers of disease progression and thereby lead to the development of better therapeutic options through pharmacogenomics. However, much more dissections into the causative pathways are needed to get a full picture of the etiology. Over the past decade, advances in research on the genetics of FTLD have revealed many pathogenic mutations leading to different clinical manifestations of the disease. This review discusses the current concepts and recent advances in our understanding of the genetics of FTLD.

## Introduction

Frontotemporal lobar degeneration (FTLD) is a heterogeneous group of dementia syndromes characterized by the circumscribed atrophy of pre-frontal and anterior temporal lobes. It accounts for 5–15% of all dementia and represents the second most common cause of dementia in patients with presenile onset.[1] Its symptoms are characterized by gradual, progressive changes in behavior, personality, and/or language with early preservation of memory [Table 1].[2]

**Table 1:** Features of FTLD

Nature of manifestation	Insidious onset and gradual progression
Age at onset	Variable and ranges from 21–85 years
Sex distribution	Equal incidence
Duration of illness	8 years (3 years in FTD-MND)
Family History	Present in 50%
Common clinical manifestation	Behavioral change (disinhibition, apathy, and stereotypic behaviors)
Clinical subtypes	FTD (70%), PNFA (10%), SD (15%)
Microscopic features	Microvacuolation, Neuronal loss, and Gliosis
Neuropathological subtypes	FTLD-tau and FTLD-U
Cognitive features	Executive deficits, change in speech and language
Neurological signs	Parkinsonism or MND
Neuroimaging	Symmetric atrophy of pre-frontal and anterior temporal lobes
Genetic Linkage	Chromosome 17q21, 17q24, 9p, 9q, and 3 with 7 loci
Genes identified	*MAPT, PGRN*, VCP, CHMP2B
Environmental Risk factors	Family history, trauma, thyroid disease, psychiatric illness etc.

FTD-MND- Frontotemporal dementia-Motor Neuron Disease, PNFA-Primary Non Fluent Aphasia, SD-Semantic Dementia, FTLD-U-Frontotemporal Lobar Degeneration with Ubiquitin positive inclusions, *MAPT*-Microtubule-Associated Protein Tau, *PGRN*-Progranulin, VCP-Valosin Containing Protein, CHMB2BCharged Multi-Vesicular protein 2B

FTLD usually segregates as an autosomal dominant trait.[3] Conventional linkage studies have identified seven chromosomal loci and a number of genes, including microtubule-associated protein tau (*MAPT*), progranulin (*PGRN*), valosin containing protein (VCP), and charged multivesicular body protein 2B (CHMP2B) associated with the disease.

FTLD has three clinical subtypes–Frontotemporal dementia (FTD), Progressive nonfluent aphasia (PNFA), and Semantic dementia (SD). Based on immunohistochemical studies, FTLD is categorized mainly into two subtypes: FTLD-tau (with inclusions of hyperphosphorylated tau as neurofibrillary tangles (NFTs)[4] and FTLD-U (with ubiquitin immunoreactive neuronal inclusions[5]) [Table 2].

**Table 2:** Clinical, neuropathological, and genetic characteristics of frontotemporal lobar degeneration

Clinical features	Chromosomal location	Name of gene	Frontotemporal lobar degeneration Neuropathology
FTDP-17	17q21.32	*MAPT*	Neuronal and glial tau positive inclusions
FTLD-U	17q21.32	*PGRN*	Ubiquitinated-TDP-43 positive NCIs, NIIs and DNs
FTLD-3	3p11.2	CHMP2B	Ubiquitinated TDP-43 negative NCIs
IBMPFD	9p13.3	VCP	Ubiquitinated TDP-43 positive NIIs and DNs
FTD-ALS	3p12	CHMP2B	No distinctive features

NCIs-Neuronal cytoplasmic inclusions; NIIs- neuronal intranuclear inclusions; DNs-Dystrophic neuritis.

## Frontotemporal Dementia

**Figure 1:**
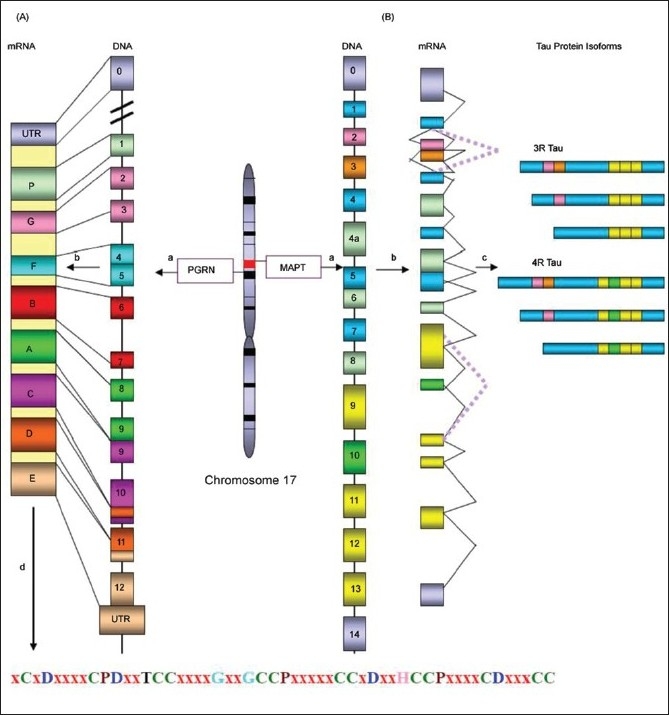
Schematic representation of the chromosomal location, genomic, and protein structures of *PGRN* and *MAPT*: (A) Structure of *PGRN* gene: Human *PGRN* located on chromosome 17 consists of 13 exons (1 noncoding and 12 coding exons). All coding exons are transcribed into mRNA, which on translation produces a full length secreted precursor protein comprised of 7.5 tandem repeats of 12 cysteinyl granulin motifs, separated by linker sequences. It is cleaved into paragranulin (P) and granulins (A-G) by elastases. (A) Structure of MAPT: Alternative splicing of *MAPT* generates six different tau isoforms by splicing in and out exons 2 and 3 in the N-terminal domain and exon 10 in the C-terminal domain, which results in 4R and 3R tau, respectively, named as 2N4R(441aa), 1N4R(412aa), 0N4R (383aa) 2N3R(410aa), 1N3R(381aa), 0N3R(352aa). a) Genomic structure, b) Transcription, c) Translation and Alternative splicing of *MAPT* gene, d) Translation of *PGRN* mRNA into progranulin protein

FTD is the most common clinical manifestation of FTLD. FTD is genetically complex and can present as familial or sporadic disorder. Most familial FTD cases are linked to chromosome 17q21. In 1994, linkage of the autosomal dominant inherited form of FTD with parkinsonism on chromosome 17q21.2 (FTDP-17) was identified and in 1997 a consensus conference introduced the term FTDP-17 to describe the patients. Subsequently in 1998, the first mutations associated with FTDP-17 were identified in gene encoding the *MAPT* protein.[6–8] This finding was of great importance since it highlights the fact that mutations in *MAPT* gene alone are sufficient to cause neurodegeneration. However, the locus heterogeneity at 17q21 was recognized with the identification of a second candidate gene for FTD named PGRN. In 2006, mutations in this gene located 1.7 Mb centromeric to *MAPT* were identified as a major cause of FTLD-U.[9,10] The identification of *MAPT* and PGRN as the two most relevant genes involved in the etiology has provided important new insights into the molecular understanding of FTLD [Figure 1].

## Genetics of FTDP-17

FTDP-17 is the prototypical tauopathy. The main pathological hallmark of FTDP-17 is the presence of neuronal and/or glial NFTs consisting of hyperphosphorylated tau proteins.[11] *MAPT* mutations are the one and only well confirmed genetic defect associated with FTDP-17.

## Microtubule-Associated Protein Tau

A direct link between neuropathology and genetic defect in FTLD was established with the discovery of *MAPT* mutations. *MAPT* mutations account for ~5–10% of the familial FTD cases. About 42 pathogenic *MAPT* mutations have been reported worldwide in a total of 125 families.[12]

**Figure 2:**
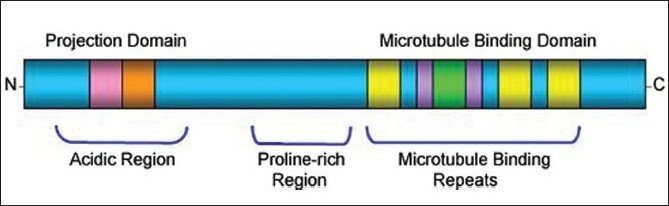
Schematic representation of functional domains of longest tau isoform (2N4R): The N-terminal projection domain is highly acidic and is followed by proline-rich region that interact with the cytoskeletal elements and plasma membrane to determine the spacing between microtubules in axons and signal transduction. C-terminal microtubule binding domain harbor microtubule binding repeats, pseudorepeats and C-terminal end and appears to regulate the polymerization and stabilization of microtubules

Human *MAPT* gene comprises of 16 exons spanning a region of more than 100 kb.[13] Tau proteins play a fundamental role in binding and stabilization of microtubules, promoting their polymerization, and thereby mediating the axonal transport.[14] In the adult human brain, alternative splicing of exons 2, 3 and 10 produces six isoforms.[15] Alternative splicing of exons 2 and 3 result in 3R and 4R isoforms with zero (0N), one (1N), or two (2N) aminoterminal inserts that mediates the interaction of microtubules with plasma membrane. Similarly, alternate splicing of exon 10 results in two tau isoforms with either 3 repeat (3R) or four repeat (4R) domains. The functional role of tau in stabilizing the microtubules resides in the C-terminal part, which harbors either 3R or 4R repeats. The binding affinities for microtubules are different for 3R and 4R tau isoforms.[16] 4R tau seems to bind and stabilize microtubules more efficiently than 3R tau, partly explained by the presence of additional binding repeat [Figure 2].[17]

Phosphorylation is the major post-translational modification of tau proteins, which appears to be developmentally regulated; fetal tau is more phosphorylated than adult tau.[18] Similarly, the expression of 3R and 4R tau is developmentally controlled with 3R tau expression in fetal brain only, but near equal amounts of 3R and 4R in adult brain. This points to the fact that regulation of tau expression is important for the function in controlling the microtubule dynamics during development.[12]

Most of the coding region mutations in *MAPT* are located on exons 1, 9, 10, 11, 12, and 13 and intronic mutations located close to the splice donor site of the intron 10, where they are supposed to destabilize a predicted RNA stem loop structure and thereby increasing the production of 4R tau.[7,11,19] The spectra of mutations in *MAPT* include missense and silent mutations along with single codon deletions and insertions. There appears a correlation between the location of mutation, cellular pathology, isoform composition and morphology of tau filaments[12] [Table 3]. Different mutations in *MAPT* will contribute different morphological and biochemical characteristics to the tau filaments observed in the pathological inclusions of FTDP-17.[12] Clinical heterogeneity has also been demonstrated for several mutations, indicating the role of both environmental and other genetic risk factors.

**Table 3:** Examples for the correlation between genotype-phenotype in frontotemporal lobar degeneration

Location of *MAPT* mutation	Predominant isoform composition	Cellular pathology	Morphology of filaments	Clinical presentation
Exon10	4R	Neuronal and Glial	Twisted ribbon-like filaments	FTDP-17
				PSP
				CBD
Intron 10	4R	Neuronal and Glial	Wide-twisted ribbons	FTDP-17
				PSP
				CBD
Missense mutations outside Exon10	3R	Neuronal	PHFs and SFs	FTDP-17

PHFs-Paired helical filaments; SD-Semantic dementia; FTDP-17-Frontotemporal dementia with parkinsonism linked to chromosome 17; PSP-Progressive supranuclear palsy; CBD-Corticobasal degeneration.

In light of their functional effects, *MAPT* mutations are classified into two groups, those modifying the microtubule interactions (mutations clustered in or near exons 1, 9, 11-13,[12]) and those affecting the alternative splicing of exon 10 (thereby disrupting the normal ratio of 3R:4R tau[7,11]). Most of the mutations affecting exon 10 and intron 10 alter the regulation of exon 10 splicing with inclusion into the tau mRNA and thereby increasing the 4R:3R ratio. Since 4R tau appears to aggregate more readily than 3R tau, overproduction of 4R tau ultimately results in cell death.

The abnormally phosphorylated pattern of tau found in the pathologic inclusions in neurodegenerative tauopathies indicates that site-specific phosphorylation apparently modulates the function and intracellular localization of tau. Various kinases and phosphatases are involved in the dynamic modulation of tau function.[20] Any imbalance in this modulation is thought sufficient to result in a pathological change, ultimately resulting in cell death.

## Genetics of FTLD-U

For the past four years, after the identification of first mutations in *PGRN*, they form the common genetic etiology associated with familial FTLD-U cases. However, genetic linkage studies have described rare, additional genetic loci associated with FTLD-U. Among them, the genetic defect on chromosomes 9p is the only confirmed loci for which the culprit gene remains to be elucidated [Table 2].

## Progranulin

Mutation data regarding the genetics of FTLD suggest that mutations in *PGRN* account for ~5–10% of all FTLD cases.[9,10] *PGRN* protein is a widely expressed, multifunctional, high molecular weight secreted growth factor, which plays significant roles in development, tumorigenesis, wound repair, and inflammation by activating signaling pathways that control cell cycle progression and cell motility.[21] *PGRN*, located on chromosome 17q21 is composed of one noncoding and 12 coding exons,[22,23] and it encodes a 593 amino acid precursor protein having a signal peptide followed by 7.5 tandem repeats of 12 cysteinyl granulin (GRN) motifs.[24] The secreted *PGRN* is subjected to proteolysis by extracellular proteases like elastase that cleave *PGRN* to generate GRNs, a process regulated by secretory leukocyte protease inhibitor (SLPI).[24,25] In periphery, *PGRN* and GRNs regulate the inflammatory cascade through opposing effects, *PGRN* being anti-inflammatory while GRNs are pro-inflammatory.[21,26] The exact function of *PGRN* in CNS is not well understood. However, it is thought to be involved in neurotrophic activity and neuroinflammation.[21]

Initially identified mutations in *PGRN* include nonsense, frameshift, and splice site mutations in three most significantly linked FTLD-U in Dutch,[10] Canadian,[9,10] and Belgian[10] families, respectively. Until date, more than 60 different pathogenic mutations are known worldwide in a total of 163 families.[27] Among these, the most frequently occurring *PGRN* mutations associated with hereditary FTD are Arg493X in exon 11, identified in thirty genealogically unrelated FTLD families worldwide[28] and Leu271LeufsX10 in exon 7 identified in 27 genealogically unrelated families.[10] Almost all pathogenic *PGRN* mutations including heterozygous deletions identified to date cause the disease by a uniform disease mechanism, creating functional null alleles that results in loss of function of *PGRN* or haploinsufficiency through nonsense mediated decay.[9,10,29]

However, a limited number of mutations do not create null alleles, instead they lead to the retention and degradation of the unspliced transcript within the nucleus, preventing the translation of *PGRN*,[9,10,29] ultimately resulting in the reduction of secreted *PGRN*. Nevertheless, the ubiquitinated neuronal inclusions in the affected regions of brain do not show immunoreactivity towards *PGRN*.[9,10]

About 70–90% patients with *PGRN* mutations show a positive family history for dementia or parkinsonism and they account for only a few incidences of sporadic FTD.[30–33] *PGRN* mutation carriers display clinical heterogeneity, FTD being the most frequent followed by PNFA. It is evidenced that noncoding genetic variability in *PGRN* can affect the disease onset and progression in FTLD-U and amyotrophic lateral sclerosis (ALS). Pathogenic *PGRN* mutations were also described in other neurodegenerative diseases like corticobasal degeneration (CBD), Alzheimer’s disease (AD), Parkinson’s disease (PD), and ALS suggesting that it is an important factor involved in general neurodegeneration.

The neuronal inclusions in FTD-U families with *PGRN* mutations are characterized by the presence of ubiquitin immunoreactive neuronal cytoplasmic inclusions (NCIs), neuronal intranuclear inclusions (NIIs), and dystrophic neurites (DNs).[32] Among these, some NIIs show a characteristic, lentiform morphology.[34] Now, the biochemical composition of the ubiquitinated NCIs, NIIs, and DNs in familial FTD-U was demonstrated as TDP-43 (trans-active response element DNA-binding protein).[35,36]

## TDP-43

**Figure 3:**
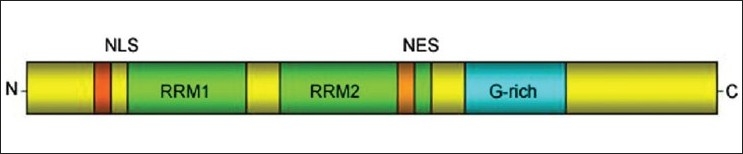
Schematic diagram of TDP-43 protein with functional domains: It contains two RNA recognition motifs (RRM 1 and 2) able to bind UG repeats in RNA as well as a glycine-rich C-terminal sequence. These features may be essential for TDP-43 to carry out its role in exon skipping and splicing inhibitory activity, which requires the C-terminal domain of TDP-43 through interaction with several members of the heterogeneous nuclear ribonucleoprotein family associated with mRNA processing. NLS-Nuclear localization sequence, NES-Nuclear export sequence

TDP-43 is a highly conserved, ubiquitously expressed nuclear protein encoded by the TAR DNA-binding protein (TARDBP) gene on chromosome 1 [Figure 3]. The normal function of TDP-43 in neurons remains to be established. However, it seems to bind DNA, RNA, and protein and has been implicated in transcription regulation.[37] Loss of TDP-43 function results in dysmorphic nucleus, misregulation of cell cycle, and apoptosis by upregulating cdk6, resulting in elevated phosphorylation of retinoblastoma protein (pRb) and related protein pRb/p130.[38]

Currently, TDP-43 is regarded as a pathogenic substrate linking a variety of distinct patterns of FTLD-U pathology including, sporadic FTLD-U with and without MND, familial FTLD-U with *PGRN* and VCP mutations as well as sporadic and familial ALS [Table 4].[39] FTLD-U is pathologically characterized by the accumulation of insoluble, proteolytically processed (with disease-specific bands at ~25kDa, ~45kda and 43kda), abnormally phosphorylated and ubiquitinated TDP-43 inclusions, accompanied by a dramatic change in the subcellular distribution of TDP-43 with the absence of the normal pattern of nuclear staining in the characteristic inclusion bearing cells.[35] Normal TDP-43 protein appears to continuously shuttle between the nucleus and the cytoplasm. Restriction of this normal nuclear shuttling may predispose the abovementioned pathological changes resulting in a loss of physiologic function and/or a toxic gain of function.[40]

**Table 4:** Neuropathological subtypes of frontotemporal lobar degeneration-U showing heterogeneous TDP-43 pathology

	Type 1 (27%)	Type 2 (42%)	Type 3 (32%)	Type 4 (rare)
Pathology	Predominant neurites	Predominant NCIs	Small neurite and NCIs	Numerous NIIS and small neurites
Clinical symptom	SD	FTD/ FTD-MND	FTD/PNFA	IBMPFD
Distribution	Superficial cortical layers	Superficial and deep Cortical layers	Superficial cortical layers	Layer II of cerebral cortex
Glial pathology	Absent-rare	Moderate-frequent	Moderate-frequent	Absent
Genetic loci	Not yet identified	9p linkage	*PGRN*	VCP

SD-Semantic dementia; PNFA-Progressive nonfluent aphasia; FTD-Frontotemporal dementia; MND-Motor Neuron Disease; *PGRN*-progranulin; VCP-Valosin-containing protein; IBMPFD-Inclusion body myopathy and Paget’s disease of bone; NCIs- intraneuronal cytoplasmic inclusions; NIIs-neuronal intranuclear inclusions.

The pathogenetic link between *PGRN* mutations and TDP-43 inclusions in FTLD-U has been explained as, loss of *PGRN* mediates caspase activation and subsequent pathological proteolytic processing of TDP-43.[41] However, other *in vitro* as well as *in vivo* studies failed to support this hypothesis and showed that caspases are not sufficiently activated by *PGRN* protein reduction that allows the TDP-43 processing in cultured cells. But it can be proteolytically processed by caspases upon induction of apoptosis that generates disease specific inclusions independent of the *PGRN* protein levels.[42]

A search for mutations in TARDBP failed to identify any pathogenic mutations in familial or sporadic FTLD suggesting that the pathological accumulation of TDP-43 might be a consequence of different underlying disease mechanisms.

## Valosin Containing Protein

FTD associated with inclusion body myopathy and Paget’s disease of bone (IBMPFD) is a rare multi-system disorder linked to chromosome 9p21-12. The gene responsible was identified as VCP, which acts as a molecular chaperone associated with several cellular functions including ubiquitin-dependent protein degradation, cell cycle regulation and apoptosis.[43] Most of the VCP mutations are located within or near the ubiquitin-binding domain.[43] Although VCP mutations have not been reported in the pathogenesis of prototypical FTLD, the neuropathology associated is a unique subtype of FTLD-U with numerous lentiform NIIs and DNs.[44] Similar to other FTLD-U subtypes, TDP-43 is the major protein constituent in these inclusions.[45]

## Charged Multi-Vesicular protein 2B

Genome-wide linkage analysis in a large dementia family originating from the Jutland region of Denmark revealed a novel locus on chromosome 3p11 and subsequently in 2004, a complex mutation in the CHMP2B gene on chromosome 3p11 was shown to segregate with affected members of the family.[46] The protein encoded by this gene is a component of the endosomal secretory complex required for transport (ESCRT) type III.[46,47] The disease segregating mutation results in aberrant splicing of exon 6, producing a truncated protein.[46] Another C-terminal truncation mutation in exon 5 was identified in a Belgium patient.[48] No other families have been found with a segregating CHMP2B mutation till date making them a rare association with FTLD. The carriers of CHMP2B mutations are characterized by ubiquitinated inclusions negative for TDP-43.[49] Dysfunction of the ESCRT results in the inability of multi-vesicular bodies to internalize membrane bound cargoes, leading to distorted endosomes and reduced protein turnover.[50] Besides FTD, CHMP2B mutations can also cause additional phenotypes including ALS and FTD-ALS.[51]

## Sporadic FTLD

Sporadic FTLD accounts for ~40% of total FTLD cases. Neuropathologically, they can be divided into neuronal and glial tau inclusions in the absence of mutations in *MAPT*,[52] TDP-43-positive FTLD-U,[39] TDP-43-negative FTLD-U and finally dementia lacking distincitive histology (DLDH).[53]

## Genetics of atypical FTLD-U

A few cases of FTLD-U were found to be ubiquitin positive but TDP-43 negative, and these cases were termed as atypical FTLD-U.[54] Recently, majority of atypical FTLD-U cases were classified under a new class termed as FUS proteinopathies, which include, FTLD-FUS, FTLD-FUS(NIFID), and FTLD-FUS(BIBD).[55] FUS (fused in sarcoma) gene is located on chromosome 16 encoding a ubiquitously expressed protein capable of binding to DNA and RNA like TDP-43.[55] FUS gene mutations were initially identified as the cause of familial ALS.[56] Subsequently, FUS immunoreactivity have been described in the ubiquitinated inclusions of atypical FTLD-U, and in subtypes of FTLD like, neuronal intermediate filament inclusion disease (NIFID) and basophilic inclusion body disease (BIBD) without any identified mutations.[55] The only remaining FTLD-U cases with yet unidentified ubiquitinated protein inclusions belong to the hereditary form of FTD-3 with mutations in CHMP2B gene.

## Genetic risk factors for FTD

Currently, there is no consistently replicated genetic risk factor linked with FTLD. The outcome of association studies of APOE ε4 and ε2 alleles on FTLD risk remains controversial.[57] H2 haplotype of *MAPT* gene has been consistently shown to be associated with FTLD-related tauopathies such as progressive supranuclear palsy and CBD, but this association lack consensus with other clinical subtypes of FTLD.

Common genetic variants in the micro-RNA binding site of *PGRN* gene are found to significantly increase the risk for FTLD-U. TT allele variant of the SNP rs5848 located in the 3’-UTR of *PGRN* can increase the affinity for micro-RNA, miR-659 thereby suppressing the translation of the protein.[58] Variants in the promoter region of vascular endothelial growth factor (VEGF) were described as a susceptibility factor for sFTLD.[59]

Analysis of Nitric oxide synthase (NOS) gene reported several SNPs in both neuronal (C276T) and endothelial (C276T) NOS, that influence the risk for sporadic FTLD.[60,61] Identifications of several mutations in the open reading frame of ubiquitin-associated protein 1 (UBAP1) gene indicating a dysfunctional UPS in the pathogenesis makes it an excellent candidate gene for FTLD. Mutations in LRRK2 gene, responsible for autosomal-dominant forms of parkinsonism[62] has been identified in an FTLD-U patient. However, due to the reduced penetrance of this substitution mutation, the pathogenecity in FTLD is not well established.

A few studies have reported the presence rare benign polymorphisms of PSEN-1 gene in patients with FTLD.[57] One study reported two novel PSEN-1 mutations in two patients, one with a clinical diagnosis of FTD but with neuropathological diagnosis of AD and another one in AD with frontal lobe signs, recommending screening for mutations in PSEN-1 in familial FTLD cases without mutations in the known relevant genes.

A genome-wide association study in individuals with FTLD-U showing linkage to 7p21 locus identified several SNPs in Trans-membrane protein 106B (TMEM106B) gene that affect the expression levels of the protein suggesting that the TMEM106B variants may confer genetic risk for some FTLD-U cases.[63] Finally, cystatin C (CST3), a cystein protease inhibitor which act as a cofactor for neurogenesis, gene analysis in FTLD patients found higher frequency of CST3 B haplotype in *PGRN* negative FTLD patients[64] warranting the search for additional genetic risk factors in FTLD. The exact role of these proteins in the pathogenesis remains to be elucidated and their association should be replicated in larger populations.

## Genetics of FTD-ALS

ALS is commonly a sporadic disorder with ~15–20% showing a family history. FTLD and ALS belong to a clinicopathological spectrum of overlapping CNS disorders that share common clinical, neuropathological, and genetic features. Approximately, 15% of the FTD cases display symptoms of ALS (FTD-ALS) and up to 50% ALS patients show cognitive impairment (ALS-FTD). Clinically, both phenotypes can occur in the same patient or within the same family and both are histologically characterized by TDP-43 pathology.[35] Genetically, the identification of a common genetic locus for FTD and ALS on chromosome 9p suggests a molecular link between FTD and ALS. For the familial ALS-FTD cases, genetic loci were identified on chromosome 9q21-q22 and 17q24-25.

**Figure 4:**
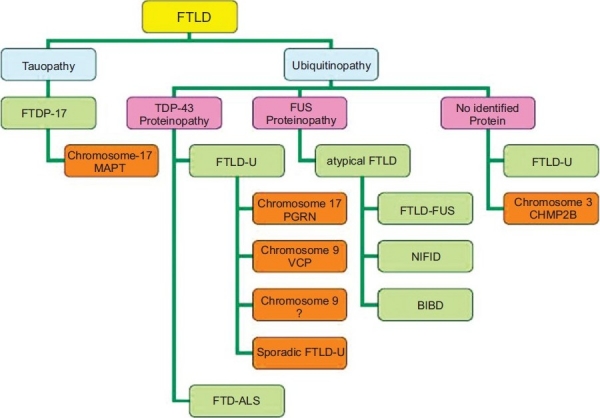
Overall view of the pathological subtypes and associated genes in frontotemporal lobar degeneration

Sequencing analysis of several genes mapped to 9p locus detected the presence of a disease segregating truncation mutation (Q342X) in the intra-flagellar transport protein 74(IFT74) in one independent American family with FTD-ALS.[65] However, no causal IFT74 mutations were identified in conclusively linked FTD-ALS families indicating additional loci (other than VCP and IFT74) for 9p linked FTD-ALS families. Moreover, sequence variations were described in *MAPT*, VEGF and dynactin1 (DCTN1) genes making them susceptibility factors for FTD-ALS. Genetic screening of TARDBP identified several pathogenic mutations located on the C-terminal region in both familial (with and without FTD) as well as sporadic ALS.[66] The overlap between these two different clinical entities suggests that the diagnostic genetic screening in FTD-ALS should include both FTD-related as well as ALS-related genes including, *PGRN*, VCP, CHMP2B, SOD1, TDP-43 etc [Figure 4].

## Conclusion

A better molecular understanding of pathogenic mechanisms in different FTLD syndromes will help find effective therapeutic strategies for the management of the disease. Future challenges in the field of genetics of FTLD include the identification of as yet unidentified genetic loci linked with FTLD, additional genetic modifying factors as well as environmental risk factors. Another challenge is to develop biomarkers in order to identify the mutation carriers, to aid the differential diagnosis, to monitor prognosis and ultimately to develop effective therapeutic strategies. Due to the unique disease mechanism, plasma *PGRN* levels may serve as a potential biomarker for the identification of carriers of *PGRN* mutation. The identification of genetic mutations in familial FTLD (particularly in *PGRN* and MAPT) has profound implications in clinical practice such as genetic counseling.
